# False Negativity of Tc-99m Labeled Sodium Phytate Bone Marrow Imaging Under the Effect of G-CSF Prescription in Aplastic Anemia: A Case Report

**DOI:** 10.22038/AOJNMB.2018.11804

**Published:** 2019

**Authors:** Akanganyira Kasenene, Aju Baidya, Chang-Yin Wang, Hai-Bo Xu

**Affiliations:** Department of Radiology and Nuclear Medicine, Zhongnan Hospital of Wuhan University, Wuhan University, Wuhan, China

**Keywords:** Aplastic anemia, Bone marrow imaging, G-CSF, SPECT

## Abstract

Granulocyte colony-stimulating factor (G-CSF) is a hematopoietic cytokine which controls the differentiation and growth of hematopoietic cells in the bone marrow. We report a severe aplastic anemia (SAA) patient with false-negative ^99m^Tc sodium phytate bone marrow imaging findings under concurrent G-CSF therapy. The first bone marrow imaging showed a normal bone marrow activity. However, the bone marrow biopsy pathology report revealed a lack of hematopoietic cells. Furthermore, the complete blood count indicated severe pancytopenia resulting in the diagnosis of aplastic anemia (AA). A second marrow scan implemented after the stoppage of G-CSF showed an abnormal bone marrow activity, which matched the pathology reports. Accordingly, the concurrent administration of G-CSF was considered as the cause of false-negative bone marrow imaging findings obtained in the first scan. Consequently, it should be kept in mind that a ^99m^Tc sodium phytate bone marrow scintigraphy during the concurrent administration of G-CSF may lead to the achievement of false negative results because it induces changes in bone marrow mimicking a normal marrow scan in patients with AA.

## Introduction

Granulocyte colony-stimulating factor (G-CSF) is a hematopoietic cytokine which controls the differentiation and growth of hematopoietic cells in the bone marrow (1). The G-CSF is often used to treat neutropenia associated with aplastic anemia (AA); therefore, it shortens the length of hospitalization. This blood growth factor affects not only neutrophils, but also mononuclear macrophages. Moreover, it influences the bone marrow imaging based on mononuclear macrophages uptake of radioactive tracer. Herein, the present study presented a severe aplastic anemia (SAA) patient case with false-negative ^99m^technetium (Tc) sodium phytate bone marrow findings under concurrent G-CSF therapy.

## Case report

A 28-year-old male presented to the hospital with a history of fatigue for 8 days, fever for 7 days, bleeding nose and gums for 6 days, and hematuria for 2 days. These signs and symptoms had no obvious cause. His past medical history and physical examination were unremarkable with the exception of patchy bleeding seen in both lower limbs. 

**Figure 1:**
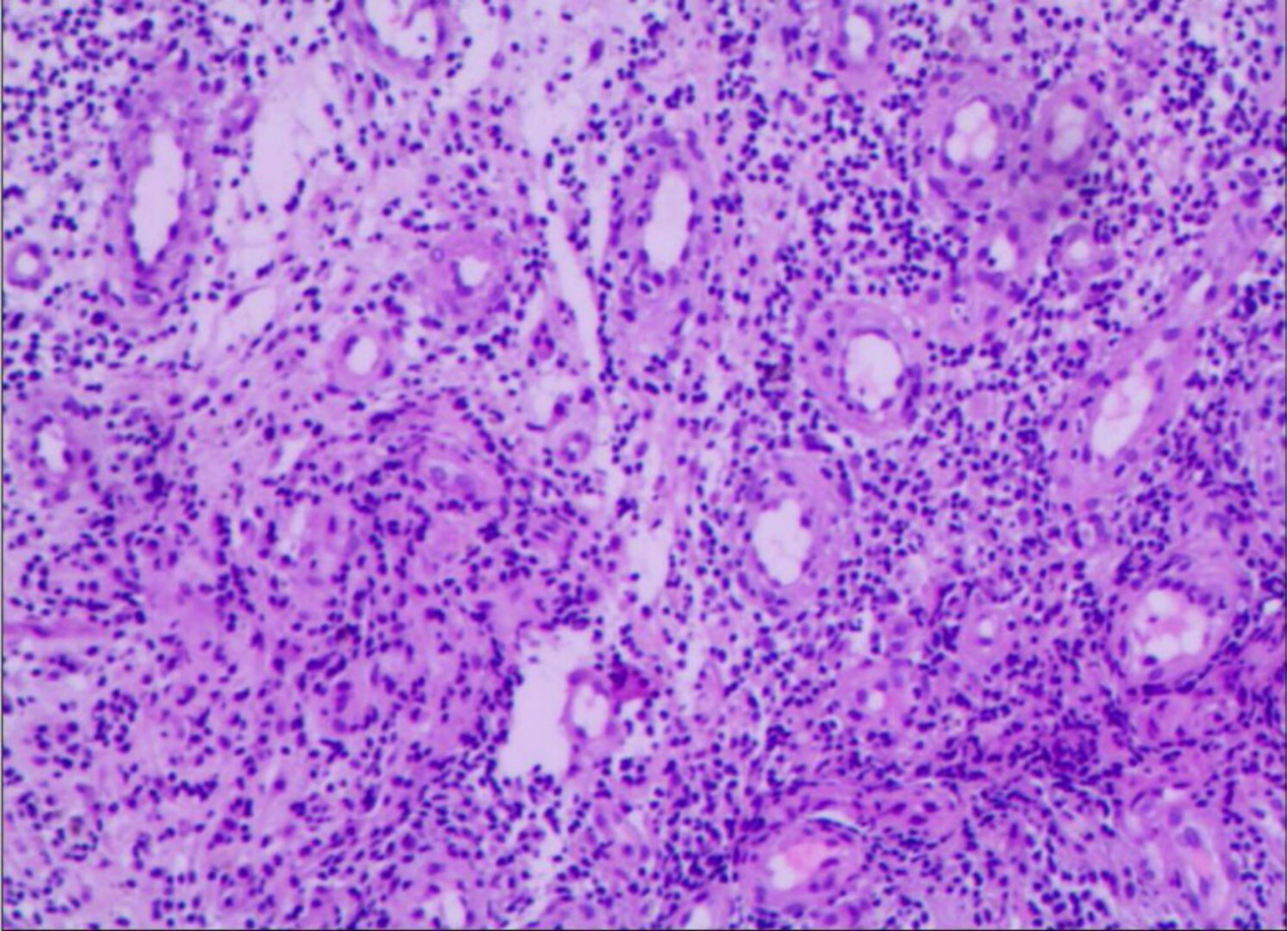
Results of bone marrow biopsy showing obviously hypocellular bone marrow spaces with increased fat spaces, in line with the aplastic anemia typical appearance (H&E×100)

On admission, the complete blood count of the patient showed severe pancytopenia (Table 1). Bone marrow biopsy demonstrated no hematopoietic cells (Figure 1); furthermore, no reticular fiber was seen (MF-0) on reticular fiber staining. Bone marrow aspiration report revealed bone marrow suppression and A

In addition bone marrow smear and T cell subset showed CD4+CD3+lymphocyte of 33.24%, CD8+CD3+lymphocyte of 24.38%, and CD4:CD8 ratio of 1.36. The results of flow cytometry were as follows: 1) low proportion of CD34+ with nucleated cells of 0.08%, 2) a significant decrease in the proportion of neutrophils and monocytes, 3) a significant reduction in the proportion of nucleated red blood cells, 4) a significant elevation in the proportion of lymphocyte, and 5) no phenotypic abnormalities. Finally, the diagnosis of SAA was made based on the patient’s history, laboratory results, and marrow biopsy and puncture.

**Figure 2:**
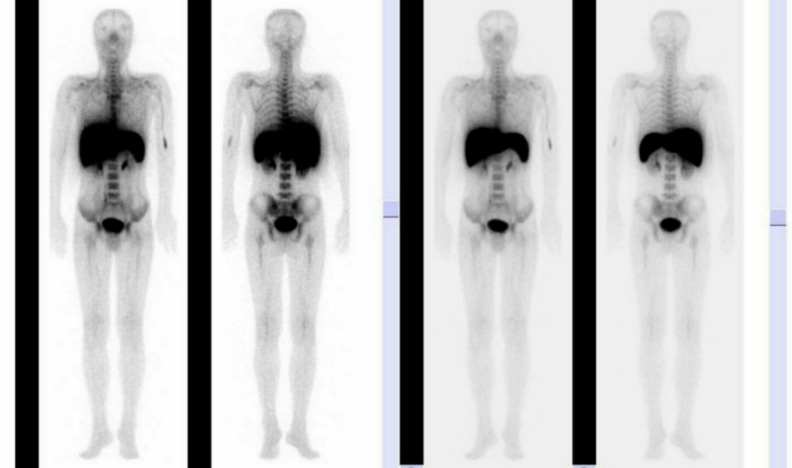
Bone marrow imaging during granulocyte colony-stimulating factor treatment showing bone marrow with a clear bone silhouette and normal bone marrow activity, correlating with level 2 of the standard bone marrow activity

The patient was put on cyclosporine and androgen for the primary disease management and G-CSF for the stimulation of hematopoiesis at Zhongnan Hospital of Wuhan University, Wuhan, China. Bone marrow imaging was performed, through single photon emission computed tomography (SPECT; E.Cam, Siemens product, Hoffman Estates, Illinois, USA) by the intravenous injection of 370MBq of ^99m^Tc-sodium phytate, 8 days after the initiation of cyclosporine plus G-CSF. The obtained images showed normal hematopoietic bone marrow activity in the central marrow correlating with level 2 of the standard bone marrow activity (Figure 2). 

**Figure 3:**
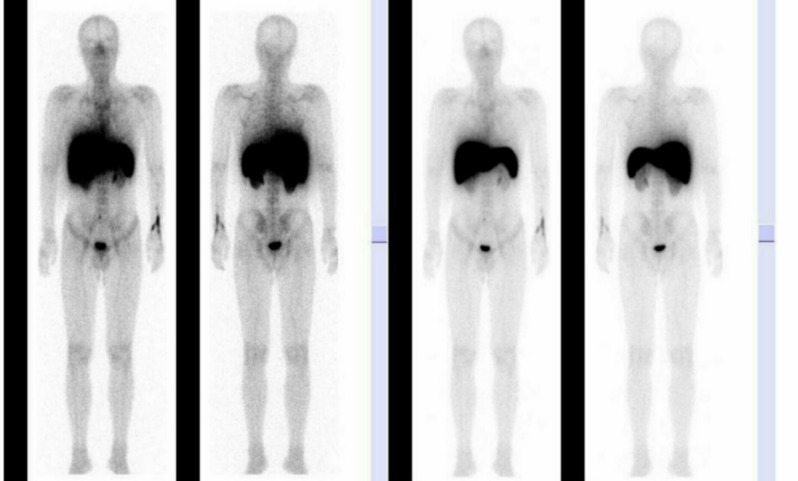
Bone marrow imaging after suspending granulocyte colony-stimulating factor treatment. Bone marrow was slightly clear and slightly higher than the peripheral soft tissues; the silhouette was not clear, indicating mild to moderate bone marrow function inhibition. These findings correlate with level 1 of the standard bone marrow activity

Table 2 presents the grading of bone marrow activity and its clinical significance in details. This marrow scan did not match the pathological and laboratory findings. The G-CSF was eventually stopped, together with the other medications. The second bone marrow scan for this patient was performed 4 months after the first marrow imaging. The result was abnormal and corresponded with level 1 of the standard bone marrow activity (Figure 3). It showed a reduction in hematopoietic bone marrow activity in the central compartment of the skeleton. The second bone marrow image was indicative of AA and matched the pathological and laboratory findings.

## Discussion

Severe aplastic anemia is a bone marrow disease in which stem cells are damaged resulting in the hematopoietic cell deficiency (1). Hematopoietic growth factors are marrow regulators that support the growth and differentiation of hematopoietic cells and the function of mature hematopoietic cells (2-4). In clinical trials, G-CSF was reported to cause a momentary increase in neutrophil count and was beneficial for the management of complicated bacterial and fungal infections in AA patients (4). 

The G-CSF promotes the proliferation, differentiation, and maturation of myeloid hematopoietic progenitor cells, and regulates the proliferation and differentiation of neutrophil cell lines. Moreover, G-CSF is a powerful stimulator and activator for monocytes and macrophages. 

**Table 1:** Complete blood count results

**Paramete**r	**Result**	**Reference range**	**Parameter**	**Result**	**Reference range**
White blood cells (×10^3^/μL)	0.88	3.9-11.1	Eosinophil (%)	0.0	0.0-8.0
Red blood cells (×10^6^/μL)	0.5	4.2-5.7	Basophil (%)	0.0	0.0-2.0
Hemoglobin (g/dL)	1.5	13.2-16.9	Absolute neutrophil (×10^3^/μL)	0.02	1.65-8.0
Hematocrit (%)	4.1	38.5-49.0	Absolute lymphocyte (×10^3^/μL)	0.66	1.0-3.5
Platelet (×10^3^/μL)	10	140-390	Absolute monocyte (×10^3^/μL)	0.00	0.04-0.9
Neutrophil (%)	2.9	38.0-80.0	Absolute eosinophil (×10^3^/μL)	0.00	0.03-0.6
Lymphocyte (%)	97.1	15.0-49.0	Absolute eosinophil (×10^3^/μL)	0.00	0-0.125
Monocyte (%)	0.0	0.0-23.0			

**Table 2:** Grading of bone marrow activity and its clinical significance (9)

**Levels**	**Image characteristics**	**Clinical significance**
0	There is no uptake of radioactive tracer in bone marrow. The radioactivity distribution of the central bone marrow is similar to that of the surrounding soft tissue.	Severe inhibition of bone marrow function
1	There is the slight uptake of radioactive tracer in bone marrow. The radioactivity of bone marrow is slightly higher than the background of the surrounding soft tissue, and the marrow outline is not clear.	Mild to moderate bone marrow suppression.
2	The bone marrow image is clear and the marrow outline is basically clear and complete.	Normal bone marrow activity
3	There is an increased uptake of radioactive tracer in bone marrow. The bone marrow image is clear, and the outline is clear and complete.	Mild enhancement of the hematopoietic activity of bone marrow
4	The bone marrow image is very clear, which is similar to bone imaging.	Significant enhancement of the hematopoietic activity of bone marrow

^99m^Tc sodium phytate is the radiotracer of bone marrow imaging in SPECT. When the tracer is injected into the body, it combines with Ca^2+^ in the blood to form a phytin colloid. ^99m^Tc phytin colloid could be absorbed by mononuclear macrophages. The administration of G-CSF significantly enhances mononuclear macrophages in the bone marrow, which signifies an increase in hematopoietic activity. Regarding this, the amount of the absorbed imaging agent reflects the hematopoietic function of the bone marrow. Therefore, these changes in the bone marrow can be detected by bone marrow scintigraphy. 

Multiple reports and case studies have defined a similar diagnostic impasse with marrow stimulation on PET imaging (5-7). The G-CSF leads to the reconversion of the fatty bone marrows to the hematopoietic marrows. This reconversion is presumably attributable to the residual hematopoietic cells stimulation in the predominantly fatty marrow (8). Differentiation of these changes without the knowledge of the bone marrow changes due to G-CSF administration might be problematic. 

Our patient’s laboratory results showed severe pancytopenia. Furthermore, the bone marrow biopsy pathology report revealed the lack of hematopoietic cells or reticular fibers, which is a typical presentation of AA. These findings did not match the first marrow scan, which was considered as a false negative result due to the effect of the concurrent administration of G-CSF. Furthermore, the follow-up scan performed 4 months after the stoppage of G-CSF matched the laboratory and pathological findings.

In conclusion, it should be kept in mind that ^99m^Tc sodium phytate bone marrow scintigraphy during the concurrent administration of G-CSF in AA patients may result in a false negative finding. Therefore, the conceptualization of the G-CSF mechanism of action within the bone marrow is a matter of significant importance for the accurate interpretation of bone marrow scintigraphy images. Radiologists and nuclear medicine physicians must be aware of any sorts of treatments the patient was on preceding any scan in order to avoid the misinterpretation of bone marrow scintigraphy images.
